# Central retinal artery occlusion and non-arteritic anterior ischemic optic neuropathy associated with an overlap syndrome: a case report

**DOI:** 10.1186/1752-1947-2-389

**Published:** 2008-12-17

**Authors:** Verônica C Lima, Tiago S Prata, Jeffrey M Liebmann, Robert Ritch

**Affiliations:** 1 Retina Service, Department of Ophthalmology, The New York Eye and Ear Infirmary, New York, NY, USA; 2 Einhorn Clinical Research Center, The New York Eye and Ear Infirmary, New York, NY, USA; 3 Glaucoma Service, Department of Ophthalmology, The New York Eye and Ear Infirmary, 310 East 14th Street, New York, NY 10003, USA; 4 Manhattan Eye, Ear and Throat Hospital, New York, NY, USA; 5 New York University School of Medicine, New York, NY, USA; 6 New York Medical College, Valhalla, NY, USA

## Abstract

**Introduction:**

An "overlap syndrome" is defined as the sequential appearance over time of two or more risk factors for glaucomatous damage. The appearance of a new risk factor can alter the course and prognosis of previously stable disease. Exfoliation syndrome is a leading cause of glaucoma and is associated with vascular disease. We report a case of central retinal artery occlusion and non-arteritic anterior ischemic optic neuropathy in a patient with overlap syndrome.

**Case presentation:**

An 87-year-old woman with longstanding stable primary open-angle glaucoma developed bilateral exfoliation syndrome, after which her intraocular pressure became uncontrolled and her glaucomatous damage progressed rapidly. She also developed ischemic arterial events in both eyes.

**Conclusion:**

The case presented here shows that overlap syndromes can lead to rapid, irreversible vision impairment. To the best of our knowledge, this is the first reported case of central retinal artery occlusion and non-arteritic anterior ischemic optic neuropathy in a patient with overlap syndrome.

## Introduction

The glaucomas comprise a group of diseases that result in a common final pathway of progressive retinal ganglion cell death, characterized by a specific pattern of optic nerve head and visual field damage. Elevated intraocular pressure (IOP) is the most important risk factor for glaucomatous damage, but is not the disease itself, as was erroneously conceptualized in the past. In primary open-angle glaucoma (POAG), no ocular findings beyond the optic disc damage and (sometimes) elevated IOP are present on examination.

Exfoliation syndrome (XFS) is an age-related disorder, which is the most common identifiable cause of open-angle glaucoma worldwide, accounting for the majority of cases in some countries [1]. XFS is characterized by the production and progressive accumulation of a fibrillar material, not only in the extracellular matrix of ocular tissues, but also in the skin and connective tissue portions of various visceral organs [2,3]. It usually presents unilaterally and when bilateral, the eyes are often markedly asymmetrically involved [4,5]. Elevated IOP occurs in about 25% of persons with XFS, and glaucoma is present in about one-third of these [6,7]. XFS has a more serious clinical course and prognosis than POAG [2]. It is more aggressive and presents with higher IOP values than POAG [5]. Its association with cardiovascular events, such as ischemic heart disease, is being increasingly described [8].

The term "overlap syndrome" has been used to describe the sequential appearance over time of two or more risk factors for glaucomatous damage and it can alter the course and prognosis of the disease [9]. Because both POAG and XFS are common, one would expect some individuals with POAG to develop XFS as an additional risk factor in older age. Nevertheless, this phenomenon has not been widely recognized or reported in the literature. We describe such a patient who developed rapid progression of glaucomatous damage and severe ocular ischemic events.

## Case presentation

An 87-year-old woman with type-2 diabetes and systemic hypertension presented with a history of POAG in both eyes (OU) for nearly 10 years. The damage was moderate and the progression rate had been relatively slow. On her initial examination, best corrected visual acuity (BCVA) was 20/30 in the right eye (OD) and 20/40 in the left eye (OS). Biomicroscopy was normal and IOP was 21 mmHg OU on antiglaucoma medications.

**Figure 1:**
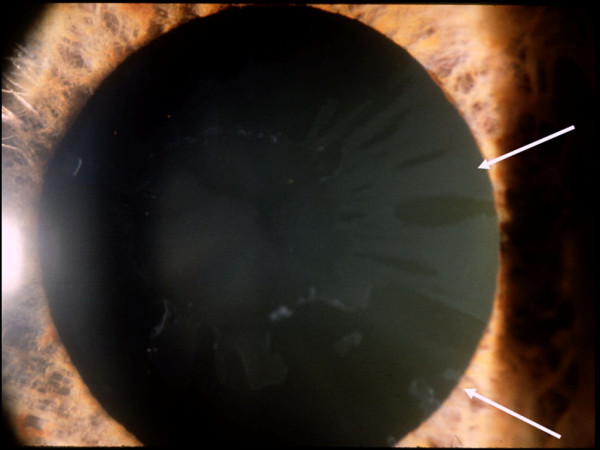
**Biomicroscopy of the left eye showing exfoliation material on the anterior lens capsule and pupillary margin (white arrows)**.

**Figure 2:**
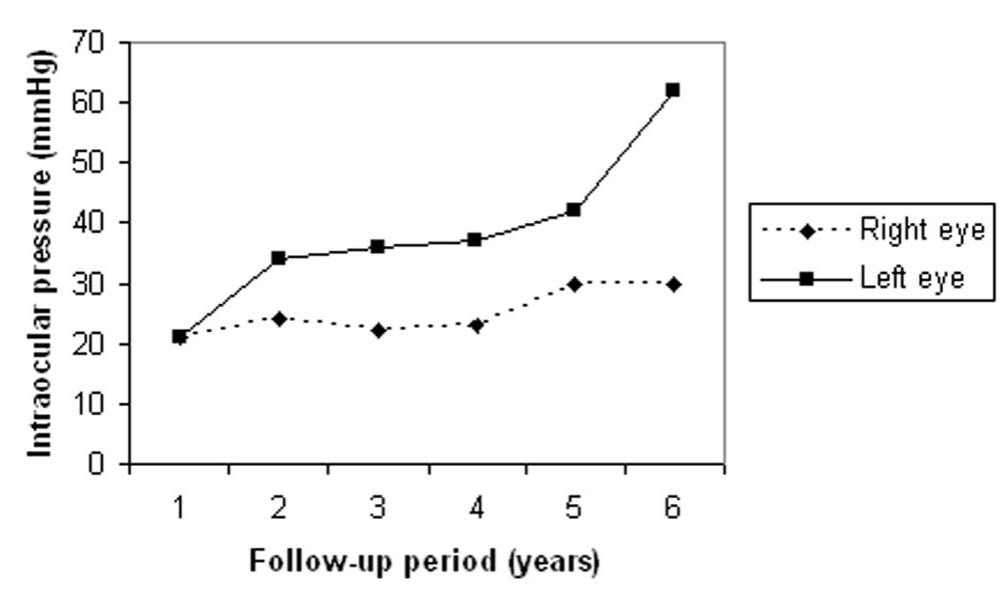
**Intraocular pressure readings for both eyes along 6 years of follow-up**.

**Figure 3:**
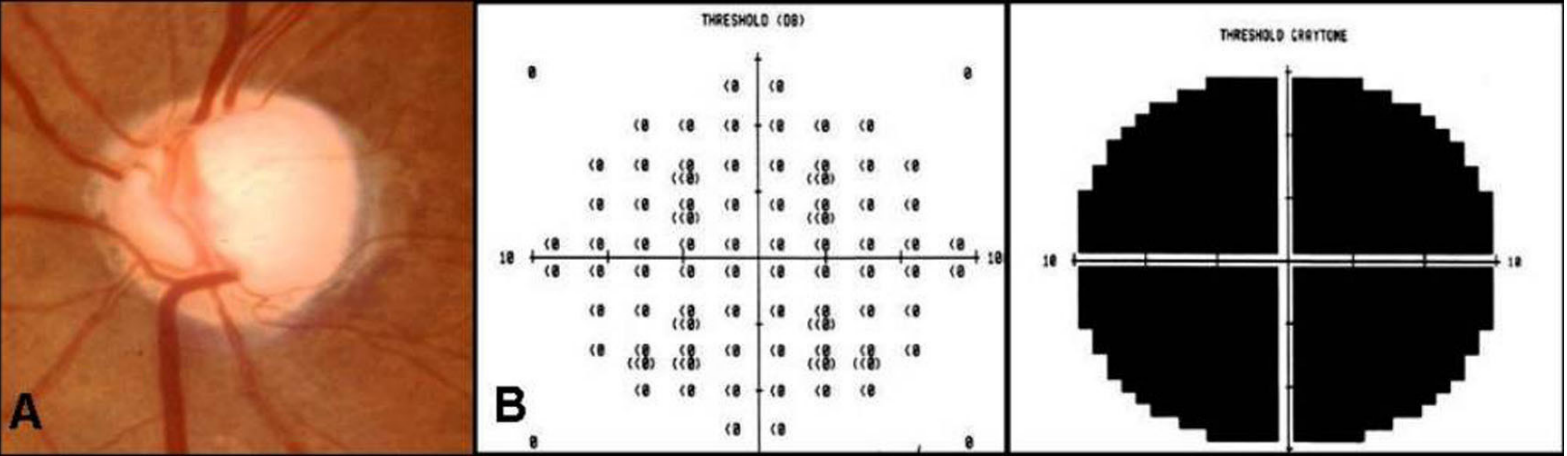
**A) Fundus picture showing an advanced glaucomatous damage with complete loss of neuroretinal rim in the left eye**. B) Central visual field test (10°) showing a complete loss of sensitivity in the same eye.

One year later, she had developed exfoliation material (XFM) on the anterior lens capsule and pupillary margin OS (Figure 1). Her IOP was 24 mmHg OD and 34 mmHg OS. Gonioscopy showed open-angles OU with visualization of XFM on the trabecular meshwork OS. Fundoscopy showed a cup-to-disc ratio of 0.5 and 0.7, respectively. This asymmetry of structural damage was attributed to the XFS onset and additional antiglaucoma topical treatment was initiated OS. Six months later, the patient complained of a marked decrease of vision OS, and a central retinal artery occlusion (CRAO) was confirmed by clinical and fluorescein angiographic examinations. Over the following 3 years, the glaucoma damage advanced OS due to uncontrolled IOP, despite treatment (Figure 2). At this time, she refused surgical or laser intervention. The OS presented an enlarged cup-to-disc ratio (0.9–1.0) and her visual acuity dropped to counting fingers at two inches (Figure 3). Several months later, XFM was noted on OD and the IOP had increased to 30 mmHg, followed by rapid glaucoma damage progression. After 2 years, cup-to-disc ratio was of 0.8 OD and 1.0 OS.

**Figure 4:**
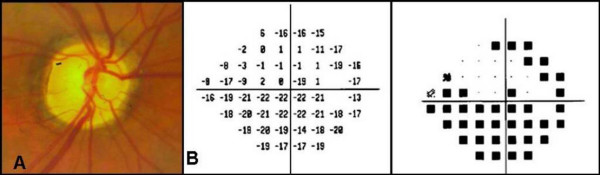
**A) Fundus picture showing a pale optic nerve head and advanced glaucomatous damage in the right eye**. B) Visual field (24°) showing a superior arcuate scotoma and a significant loss of sensitivity inferiorly in the same eye.

During the last year of follow-up, the patient developed further bilateral visual impairment. BCVA was 20/70 OD and light perception OS, and IOP was 30 mmHg OD and 62 mmHg OS. Fundus examination OD revealed a pale optic nerve head, despite no visible glaucoma structural progression, which was diagnosed as a non-arteritic anterior ischemic optic neuropathy (NAION) after clinical and visual field examinations (Figure 4).

## Discussion

Our patient had POAG in both eyes for almost 10 years. Her disease course was stable until she developed XFS, followed by rapid progression and severe ischemic ocular vascular events OU non-simultaneously. The progression of her glaucoma followed the development of XFS in each eye, associated with markedly increased IOP.

We believe that this patient can be considered as having an overlap syndrome [9]. The appearance of a new risk factor in a patient whose condition has been stable is suggested by loss of control of the IOP or acceleration of disc and visual field damage after a long period of stability. The appearance of a new disease, XFS in this case, which also leads to glaucomatous damage, altered the course and prognosis of the POAG which had been stable for almost 10 years.

The underlying risk factors beyond elevated IOP for POAG remain largely unknown. Elevated IOP is a sign of trabecular dysfunction and increased aqueous outflow resistance, but events at the genetic and molecular levels predisposing the trabecular meshwork to developing elevated IOP are only just beginning to be elaborated. Mutations in the gene coding for myocilin have been best described. The gene coding for optineurin has been sequenced and associated with a minority of patients with normal-tension glaucoma. Other loci have been identified, but the associated genes have not yet been identified. Presumably, many different genes will eventually be discovered to be causative. Nonetheless, these abnormalities constitute risk factors for the development of POAG. In the same way, recent advances in the understanding of the pathogenesis of XFS have lead to the discovery of a disease-related genotype that could help explain the systemic and ocular findings in these patients. Briefly, the lysyl oxidase-like 1 (LOXL-1) gene snip-out could be responsible along with other genes for the aging process of different body tissues, including the skin, brain, myocardium and vascular endothelium.

An increasing number of reports have demonstrated an association between XFS and systemic vascular events, including hypertension, angina, myocardial infarction and stroke [8,10]. Elevated plasma homocysteine levels, a risk factor for cardiovascular disease, are more common in XFS and XFG patients than in healthy controls [11]. XFS is associated with branch and central retinal vein occlusion [12,13]. Hyperhomocysteinemia is associated with central retinal vein occlusion, NAION and CRAO [14,15]. After developing XFS, our patient experienced two serious ocular arterial occlusive events, respecting the laterality of the XFS onset. We understand that all of the features noted in this case reinforce the hypothesis that the development of an overlap syndrome was associated with the onset of the ocular vascular events. To the best of our knowledge, this is the first reported case of CRAO and NAION in a patient with overlap syndrome.

It is important to stress some clinical implications of this case report. First, since XFS is yet untreatable and seems to be an additional risk factor for vascular complications in older patients, it is important to have strict clinical control of the other systemic comorbidities associated with cardiovascular diseases, such as diabetes and systemic hypertension. Second, if a POAG patient develops XFS, careful follow-up is advised, since the disease progression could be rapid. It is known that the amount of visible XFM in the anterior chamber may vary significantly in eyes with XFS. Therefore, it is very important to perform a careful examination of any POAG patient presenting with progressive IOP increase despite current treatment, in order to rule out a possible overlap syndrome. Finally, although presenting with an initial asymmetric onset, XFS can severely affect both eyes. Thus, close monitoring of the fellow-eye is important in clinical unilateral cases.

## Conclusion

Overlap syndrome (described here as a combination of POAG and XFS) might lead to a rapidly and irreversible vision impairment due to glaucoma damage progression. Moreover, it can be accompanied by other ocular comorbidities, such as vascular occlusive events, which can also threaten vision function.

## Abbreviations

IOP: intraocular pressure; POAG: primary open-angle glaucoma; XFS: exfoliation syndrome; BCVA: best corrected visual acuity; OU: in both eyes; OD: in the right eye; OS: in the left eye; XFM: exfoliation material; CRAO: central retinal artery occlusion; NAION: non-arteritic anterior ischemic optic neuropathy; LOXL-1: lysyl oxidase-like 1

## Consent

Written informed consent was obtained from the patient for publication of this case report and accompanying images. A copy of the written consent is available for review by the Editor-in-Chief of this journal.

## Competing interests

The authors declare that they have no competing interests.

## Authors' contributions

VCL has made substantial contributions to acquisition of data, conception and design of the manuscript; TSP has been involved in the data acquisition and drafting and revising the manuscript critically for important intellectual content; RR and JML have made substantial contributions to conception and design, they have been involved in drafting the manuscript and revising it critically for important intellectual content, and they have given final approval of the version to be published.
